# Estimation and correction of non-specific binding in a large-scale spike-in experiment

**DOI:** 10.1186/gb-2007-8-6-r126

**Published:** 2007-06-26

**Authors:** Eugene F Schuster, Eric Blanc, Linda Partridge, Janet M Thornton

**Affiliations:** 1 European Bioinformatics Institute, Wellcome Trust Genome Campus, Hinxton Cambridge CB10 1SD, UK; 2 MRC Centre for Developmental Neurobiology, King's College London, Guy's Hospital Campus, London SE1 1UL, UK; 3 Department of Biology, University College London, Darwin Building, Gower Street, London WC1E 6BT, UK

## Abstract

**Background:**

The availability of a recently published large-scale spike-in microarray dataset helps us to understand the influence of probe sequence in non-specific binding (NSB) signal and enables the benchmarking of several models for the estimation of NSB. In a typical microarray experiment using Affymetrix whole genome chips, 30% to 50% of the probes will apparently have absent target transcripts and show only NSB signal, and these probes can have significant repercussions for normalization and the statistical analysis of the data if NSB is not estimated correctly.

**Results:**

We have found that the MAS5 perfect match-mismatch (PM-MM) model is a poor model for estimation of NSB, and that the Naef and Zhang sequence-based models can reasonably estimate NSB. In general, using the GC robust multi-array average, which uses Naef binding affinities, to calculate NSB (GC-NSB) outperforms other methods for detecting differential expression. However, there is an intensity dependence of the best performing methods for generating probeset expression values. At low intensity, methods using GC-NSB outperform other methods, but at medium intensity, MAS5 PM-MM methods perform best, and at high intensity, MAS5 PM-MM and Zhang's position-dependent nearest-neighbor (PDNN) methods perform best.

**Conclusion:**

A combined statistical analysis using the MAS5 PM-MM, GC-NSB and PDNN methods to generate probeset values results in an improved ability to detect differential expression and estimates of false discovery rates compared with the individual methods. Additional improvements in detecting differential expression can be achieved by a strict elimination of empty probesets before normalization. However, there are still large gaps in our understanding of the Affymetrix GeneChip technology, and additional large-scale datasets, in which the concentration of each transcript is known, need to be produced before better models of specific binding can be created.

## Abstract 2

A combined statistical analysis using the MAS5 PM-MM, GC-NSB and PDNN methods to generate probeset values from microarray data results in an improved ability to detect differential expression and estimates of false discovery rates compared with the individual methods.

## Background

Despite the ubiquitous use of Affymetrix GeneChip arrays (Affymetrix has recorded more than 3,600 publications with data collected on this platform), we have a limited understanding of the technology. The physico-chemical details of hybridization of target mRNA on these arrays are still incomplete and models for specific and non-specific DNA-RNA interactions are continuously being refined (a recent example can be found in [1]). A deeper understanding of these processes is required to better separate experimental variation from biological variation. For example, it would allow for addressing the influence of the amount of labeled RNA on the intensity of the probes that do not specifically bind any transcript in the RNA sample. The removal of non-specific signal will lead to improvements in normalization, and it may also lead to more effective normalization methods, as normalization methods still suffer from some shortcomings [2].

The Affymetrix technology is remarkably simple and uniform throughout a large number of different array types: every feature on the chip contains millions of identical 25 nucleotide long DNA molecules covalently bound to the GeneChip array. Features are paired on the chip, the two members' sequences being identical except for the central (13th) nucleotide, which is changed to the complementary base in one of the members. The sequence exactly complementary to the target sequence is called PM for perfect match, while the other is called MM for mismatch. A MM probe is designed to measure the non-specific binding (NSB) of its partner PM probe. Feature pairs that probe a specific transcript are grouped into a reporter set. Depending on the GeneChip array type, reporter sets are made of 11 to 16 individual feature pairs, or reporters.

Processing raw Affymetrix expression data usually consists of three different operations on the data: the first operation is the separation of the signal due to specific hybridization of the target sequence to the probe from non-specific signal associated with a background signal from the chip surface and the non-specific binding of labeled cRNA. The second operation is the normalizing of this specific signal between experiments, and the third part is the summarizing of the signals from each probe into a synthetic expression value for the whole probeset. These different aspects of normalization may or may not be separate in the actual software implementation of the algorithm, and their order of application is not necessarily identical for different algorithms. An additional normalization at the probeset level may also improve the performance of a method.

In order to carry out a detailed analysis of the impact of the probe sequence on the observed intensity, one ideally needs a pool of mRNA where the concentration of every transcript is known. A large number of different target sequences is also required to sample the sequence space spanned by the probes. The influence of non-specific hybridization can also be studied, as various levels of target 'promiscuity' are inevitable as soon as the number of target sequences is large. Because of the huge effort required to generate such a controlled dataset, hybridization modeling and normalization calibration to date have been done on high-quality, but much smaller spike-in experiments. But recently, a larger scale dataset of known composition (the GoldenSpike dataset) has been made publicly available [3], consisting of six hybridizations, three replicates of two different cRNA compositions, hereafter called control (C) and spike-in (S), as the cRNA concentration in the latter samples are always equal to or higher than in the former samples.

Unlike other spike-in experiments in which transcripts are spiked into biological samples of unknown composition (for example, the Latin-square dataset [4]), all transcripts are known within the GoldenSpike dataset. All the cRNA samples are made of 3,859 clones of known sequence, 1,309 of which have a higher cRNA concentration in the S samples, while the cRNA concentrations of the remaining 2,550 clones are identical in all samples. The concentrations of the cRNA pools span slightly more than one order of magnitude, and the cRNA concentrations of the S samples are between one and four times larger than the corresponding clones' cRNA concentrations in the C sample.

This experimental setup represents a biological situation where roughly one-quarter of the genome is expressed, and among those expressed genes, about one-third are differentially expressed; however, compared to a 'normal' dataset, there are no 'down-regulated' clones, so the data are unusual and heavily imbalanced. This dataset provides a harsh test for normalization methods, as most of them assume a considerable degree of similarity between the mRNA concentration distribution within each experiment. The large differences in amounts of labeled cRNA in the GoldenSpike dataset violate this normalization assumption, and the effects are further increased by the absence of biological variability, as replicates are only technical.

The cRNA samples were generated from PCR products from the *Drosophila *Gene Collection (DGC release 1.0) [5]. Plates of PCR products (13 separate plates in total) were mixed into 17 pools. Each pool was labeled and added to the final cRNA sample at specific concentrations and hybridized to the Affymetrix DrosGenome1 GeneChip array. This means that the absolute concentration of an individual cRNA transcript is not known, and the concentrations of transcripts within a pool will vary greatly depending on the quality of the PCR amplification for an individual clone. However, the relative concentration between C and S samples for individual transcripts will be known and the same for every transcript within a pool. Choe and colleagues [3] used the GoldenSpike dataset to compare several algorithms commonly used in microarray analysis and developed a 'best-route' method for the normalization and statistical testing of microarray data. To avoid the problems associated with the imbalance of transcript levels in C and S samples, they normalized the data using a subset of probesets that were known to be at the same concentration in each sample. In the GoldenSpike normalization method, non-specific binding is corrected by subtracting the MM signal from its partner PM signal using the MAS5 method [6,7], and the PM-MM signals are normalized separately with the loess, quantiles [8], constant and invariantset [9] methods available in BioConductor [10] to create four separate expression measures at the probe level. The PM-MM signals within a probeset are then summarized into one expression value by both the tukey-biweight [6,7] and the medianpolish [8] summary methods to create eight different expression measures. The final step of the GoldenSpike normalization is loess normalization of the probeset expression values for each expression measure [3].

Using receiver-operator characteristics (ROC) curves, the Cyber-T method was determined to be the most sensitive for detecting fold changes and reducing false positives (FPs) compared to a *t*-test or significance analysis of microarrays (SAM [11]) method [3]. The Cyber-T method is based on the *t*-test method but uses a signal intensity-dependent standard deviation to reduce the significance of high fold changes in probesets with low signal intensity [12]. To identify a 'robust' set of probesets that exhibit differential expression, Choe *et al*. [3] also recommended a method that combines the test statistics as calculated by Cyber-T of the eight expression values methods. For multiple hypothesis testing correction, the sample label permutation method (as used in SAM) was used to estimate the number of FPs [13-15] and generate *q*-values (analogous to false discovery rates (FDRs)).

It has been suggested that there are serious problems with the GoldenSpike dataset [16]. Some of the problems are associated with using the dataset to evaluate statistical inference methods, as the distribution of *P *values for null probesets (that is, probesets with equal concentrations in C and S samples) is biased for low values and is not uniformly distributed between 0 and 1. For the GoldenSpike dataset, there is a bias for null probesets to have low *P *values, and the bias results in the calculated FDRs being much higher than the actual. We suggest that *P *value bias is partially due to the MAS5 PM-MM method to correct for non-specific binding.

Due to the high number of FPs at low intensity using MAS5 PM-MM, we were motivated to re-analyze the GoldenSpike dataset to assess the performance of the probe sequence-dependent models (the Naef [17] and Zhang [18] models). These empirical models adjust probe signal intensity based on probe sequence. For example, probes that contain many adenines tend to have lower intensity than probes with many cytosines, especially if the adenines and cytosines are in the center of the probe. We tested the ability of the models to estimate NSB of empty probesets and then used the publicly available implementations of the models to compare 300 different combinations of NSB correction/probe-level normalization/probe summary/probeset-level normalizations. Performance of each method was based mainly on the rates of finding true positives (TPs), and FPs and the estimation of FDRs. We also assessed the benefits of combining the statistical analysis of several methods.

Given that there are thousands of transcripts in the GoldenSpike dataset, we were able to expand the analysis of the data to include performance measures of methods at different intensities to detect any changes in performance for probesets with intensities dominated by NSB (empty or low intensity) and those dominated by specific-binding signal (medium and high intensity).

## Results and discussion

### Alignment of transcripts to probesets

The cRNA samples used in the GoldenSpike dataset were generated from 3,859 clones, and we were able to generate 'transcript' sequence information for 3,851 of the clones based on recent sequence information. From this information, we aligned the transcript sequences to the PM probes and found all the exact matches to PM probes. We were able to map the transcripts that had the same concentration in C and S samples, also referred to as having a fold change of 1 (FC = 1), to at least one probe within a probeset for 2,495 probesets. Spiked-in transcripts that had a higher concentration in S samples (FC > 1) were mapped to 1,284 probesets. Of the remaining probesets, 10,104 were unbound or 'empty' probesets, and 127 probesets could be mapped to multiple transcripts. For mixed probesets, 58 can be aligned to only FC = 1 transcripts and 69 can be aligned to at least one FC > 1 transcript (Additional data file 1). Choe and colleagues [3] found alignments to a similar number of probesets (2,535 FC = 1, 1,331 FC > 1, 13 mixed, and 10,131 empty).

### Greater NSB signal in spike-in samples than control samples

**Figure 1:**
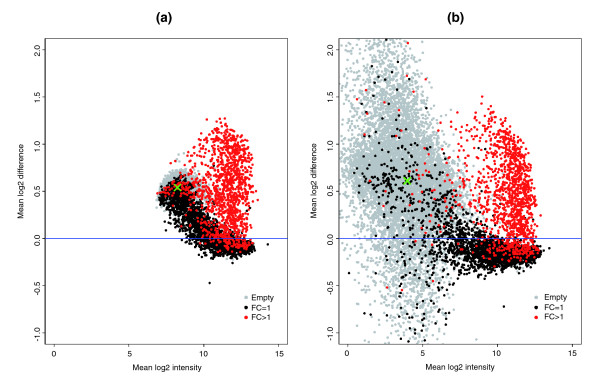
Plot of mean log2 difference versus mean log2 intensity (MA plot) of C and S samples. MA plots for the **(a) **PM-only and **(b) **MAS5 PM-MM summary methods. Log2 differences greater than 0 imply that the average log2 intensity values in S samples are greater than C samples. Grey points represent empty probesets, black points represent FC = 1 probesets, and red points represent 'differentially expressed' FC > 1 probesets. The green 'x' is located at the mean log2 difference and mean log2 intensity of empty probesets.

In the GoldenSpike dataset, there is a large difference between NSB signal in C and S samples. For un-normalized PM probes that have been summarized into probesets, empty probesets are 50% brighter in the S samples compared to the C samples (Figure 1a). The difference in NSB signal is also evident in low intensity FC = 1 and FC > 1 probesets, and we suggest this difference is due to the different amounts of labeled cRNA added to each hybridization.

The C and S samples in the GoldenSpike dataset have similar amounts of total RNA hybridized to the Affymetrix chips but have different amounts of labeled transcript. The S samples have almost twice the amount of labeled cRNA hybridized to each replicate chip as C samples (due to the 'spiked-in' transcripts). For the samples to have the same amount of total RNA hybridized, the C samples were supplemented with unlabeled poly(C) RNA. As the cRNA in the C and S samples are made from the same PCR amplification and labeling reaction, the difference in the total amount of labeled RNA hybridized to the chips is the most likely explanation for the empty and low intensity probesets in the S samples being significantly higher than in the C samples. Proper correction for NSB would result in empty and FC = 1 probesets having a log2 difference of zero between C and S replicates.

### MAS5 PM-MM is a poor model for estimating NSB

#### False positives for differentially expressed genes

The most common model for the removal of non-specific binding signal is the MAS5 PM-MM model. In this model, the MM probe intensity is an estimate for the non-specific binding of its partner PM probe. However, this model does not seem to correct for non-specific binding as the intensities of empty S probesets are still roughly 50% greater than empty probesets in C samples (Figure 1b).

**Figure 2:**
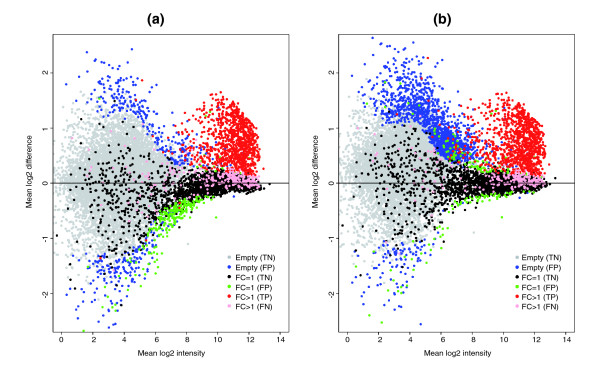
Plot of mean log2 difference versus mean log2 intensity (MA plot) showing FPs. MA plots are for probes normalized with the GoldenSpike method using **(a) **all null probesets (empty and FC = 1) as a subset and **(b) **only FC = 1 probesets as a subset. In the plots, red spots represent FC > 1 probesets that are called significantly differentially expressed (*q *< 0.1) by the modified Cyber-T method suggested by Choe *et al*. (that is, TPs). Pink spots represent FC > 1 false negatives. Grey symbols represent empty probesets that are not called significantly differentially expressed (true negatives), and blue symbols represent empty probesets that are called significantly differentially expressed (FPs). Black symbols represent FC = 1 true negatives, and green symbols represent FC = 1 FPs.

If NSB signal is not estimated correctly, then normalization can potentially distort the analysis of the data. This is clearly demonstrated by normalizing the GoldenSpike dataset with the method recommended by Choe *et al*. [3] (the GoldenSpike method) using all null probesets (empty and FC = 1) as a subset for normalization. Normalization cannot compensate for improper correction of NSB signal, and null-probeset normalization will shift the log2 difference between empty probesets towards zero, at the expense of low intensity FC = 1 probesets, which become down-regulated (Figure 2a). If only FC = 1 probesets are used as a subset for normalization, then the FC = 1 probesets behave as expected (log2 differences centered around zero), but the empty probesets are up-regulated (Figure 2b). By comparing the number of probesets with *q*-values (an estimate of FDRs) below 0.10 as calculated by the Cyber-T method recommended in Choe *et al*. [3], the total number of FPs is reduced by normalization using all null probesets compared to FC = 1 probesets, but the number of FC = 1 FPs is greater (Table 1).

**Table 1:** False positives using the GoldenSpike MAS5 PM-MM methods

	Total	Null subset normalization	FC = 1 subset normalization
Empty	10,104	487	1,729
FC = 1	2,495	251	180
FC > 1	1,284	1,015	1,057

All probesets with *q*-values below 0.10 based on the GoldenSpike normalizations and Cyber-T statistical analysis [3].

#### *P *value distributions of null probesets

The *q*-value of a probeset is defined as an estimate of the proportion of FPs among all probesets with equal or lower *q*-values. To calculate *q*-values, a test statistic is generated for the data and for permutations of the data. The permutations are based on randomly re-assigning the sample labels (for example, given the six GoldenSpike RNA samples and three C/S replicates, there are nine permutations of sample labels that do not match the 'correct' labeling), and for a particular test statistic cutoff, the mean number of probesets called significant after sample label permutation is an estimate of the number of FPs for that cutoff value and used to calculate *q*-values [11,15]. At a given test statistic, if 100 probesets are significant when the sample labels are correct and on average 10 probesets are significant when the sample labels are permuted, then the estimate of FDR for that cutoff value (*q*-value) is 0.10.

**Figure 3:**
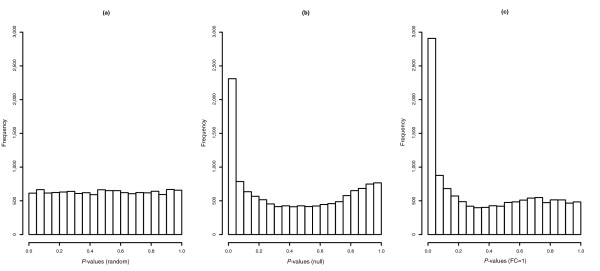
Histograms of *P *values for all null probesets. **(a) **The expected distribution of *P *values for null probesets is a uniform distribution between 0 and 1, generated at random. The observed *P *value distribution after normalization using all **(b) **null probesets as a subset and **(c) **only FC = 1 probesets as a subset are shown. MAS5 PM-MM was used for NSB correction, probes were normalized with the loess method, probes were summarized into probesets with medianpolish, and *P *values were generated with Cyber-T.

Proper estimation of *q*-values requires that null probesets have a uniform distribution of *P *values [19], but in the GoldenSpike dataset, the differences in NSB results in many null probesets having low *P *values. After NSB correction and normalization, the log2 mean difference between null probesets in C and S samples should be centered around zero and the *P *values for null probesets should be uniformly distributed between 0 and 1, but after MAS5 PM-MM correction, these requirements are not met (Figures 2 and 3). This results in *q*-values that considerably underestimate the true *q*-values [3,16], and our analysis shows that at a 0.10 *q*-value cutoff, the real *q*-value is 0.77 for FC = 1 probeset normalizations. We suggest that to reduce the number of FPs, it is essential to make a better estimate of NSB signal and/or to better detect and remove all probesets that are not bound by their target transcript. For example, if all empty probesets are removed and the *q*-values are re-calculated, then a *q*-value of 0.10 would correspond to a true *q*-value of 0.28.

While the differences in NSB signal in C and S samples account for a significant proportion of null probesets with low *P *values, there are other issues that will effect the *P *value distribution of null probesets. A single C and S sample was generated and an aliquot from each sample was used to create each replicate, and technical variation in the methods to generate each hybridization could result in subtle *P *values biases if not accounted for in the statistical analysis. For example, the mean raw PM values for FC = 1 probesets (1,898, 2,210 and 2,495 for C replicates, and 2,257, 1,803, and 2,466 for S replicates) suggests different sized aliquots and possible pairing between C and S replicates based on aliquot size. Also, most fluidics stations can only hybridize four samples at one time, and with six replicates, there might be two batches of hybridizations. It is beyond the scope of this manuscript to address all the possible sources of technical variation and account for it in statistical models, as we have concentrated on using the GoldenSpike dataset to infer the best methods to correct NSB and have not used the dataset to evaluate statistical methods. With only three replicates, it is also unlikely that technical variation can be properly taken into account. For example, analysis of the Latin Square spike-in experiment (3 replicates of 14 samples with 42 spiked-in transcripts) [4] revealed similar bias null probesets having low *P *values bias for null probesets, even when the set of TPs was expanded to include probesets that do not perfectly match the spiked-in transcripts [20].

### Probe sequence-dependent models for NSB correction

Having shown that PM-MM is a poor model for estimating NSB signal, we tested if the non-specific binding signal could be better modeled with the Zhang and Naef probe sequence-dependent models for short oligonucleotide binding. To do this, we used the GoldenSpike dataset at the level of the probes rather than at the level of probesets and took great care to align the probe sequences on the clones' sequences when available. When there was no complete clone sequence, we used the *Drosophila *Genome Release 4.0 [21] to pad the missing sequence. To reduce any effect of promiscuity, we used only empty probes that cannot be mapped to any clone, even when up to six alignment errors are considered.

We tried to evaluate the success of two models describing NSB of empty probes: a model based on Naef *et al*. [17] that assumes that the affinity of a probe can be described as the sum of the single nucleotide affinities across the probe. The second model is based on Zhang's position-dependent-nearest-neighbor (PDNN) model [18], in which the affinity of a probe can be described by the sum of all nearest-neighbor di-nucleotides within a probe, but the influence of each di-nucleotide is weighted depending on its position in the probe. Both models are described in Materials and methods.

**Figure 4:**
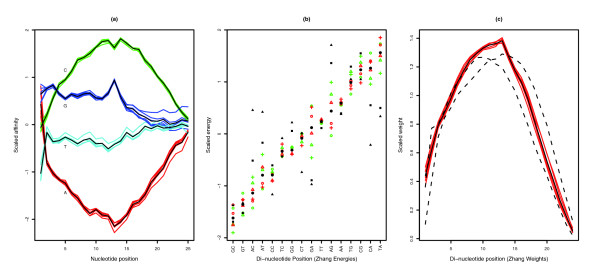
Agreement between model parameters from the six replicates. **(a)** The Naef model scaled affinity parameters. They show good consistency, except for the behavior of guanine near the probe attachment point (nucleotide position 25). **(b)** Zhang model scaled 'binding energy' parameters for each of the three control samples (red circles, triangles and crosses) and for each of the three spike-in samples (green circles, triangles and crosses) for each di-nucleotide pair. In addition, the average over the six samples is indicated with black circles and the average over the two sets of energy parameters distributed for seven chip types distributed with Perfect Match [26] is indicated with black triangles and squares. The Zhang energy parameters are not as consistent as the Naef parameters, especially for AG and GA di-nucleotides. **(c)** Zhang's weights parameters for the six experiments (red), their mean (black line) and the average of the weights for the seven sets of weights (for non-specific and specific binding) distributed with the PDNN program (dotted lines). The parameters refined here show a clear difference from the averages over the two sets of weights distributed with PDNN. In all cases, these weights confirm the importance of the central part of the probe.

Figure 4a shows that the Naef model predicts a low affinity for sequences with many adenines (A), while a sequence with many cytosines (C) would have a high affinity. Using the Naef model, fitted parameters for contributions of signal at each position of a probe show a good consistency across all six RNA samples (both C and S samples), and the model could reasonably reproduce the observed intensities of the empty probes (Table 2).

**Table 2:** Results of fits on empty probes

	C1	C2	C3	S1	S2	S3
Naef model	0.785	0.793	0.789	0.799	0.788	0.770
Zhang model	0.820	0.829	0.826	0.834	0.827	0.808
Naef scaling	0.782	0.790	0.788	0.796	0.787	0.766
Zhang scaling	0.821	0.830	0.828	0.835	0.830	0.810

The table shows the correlation coefficients between observed intensities for the empty probes on the three control (C) and spike-in (S) experiments and the corresponding model predictions. The 'model' entries correspond to the correlation between observations and predicted values for refinements of models, including the affinity, binding energy and weights parameters. The 'scaling' entries refer to the correlation between the observation from the cross-validation set and the predicted values obtained by refining restricted models, where affinities, binding energies and weights are kept constant at values obtained from the fits on the complete models (see Materials and methods). The agreement between the values of correlation coefficients from both types of refinement suggests that the affinity, binding energy and weights parameters are general and do not depend on the sequence or the experiment.

The Zhang model predicts that probes with many GC di-nucleotides would have a high signal especially if the GC di-nucleotides are in the middle of the probe, as shown in Figure 4b,c. The fitted binding energy parameters derived for each di-nucleotide in the six experiments are not as consistent as the parameters fitted in the Naef model, but the parameters fitted for the weights associated with each di-nucleotide position are more consistent and confirm the importance of the central part of the probe. Table 2 shows that the Zhang model seems to predict the observations better than the Naef model despite having fewer parameters, which apparently contradicts a previous observation that di-nucleotide binding was not the main effect in the binding [17]. However, our fitted parameters for the Zhang model were significantly different from those publicly available for four human chips and three mouse chips.

We had also planned to use the GoldenSpike dataset to investigate the specific binding signal using models derived from the Zhang and Naef models described above, taking advantage of the fact that the clones' cRNA concentrations are approximately known. Unfortunately, a detailed inspection of the data suggested that there is a very high variability between clone concentrations within a single PCR pool and we were not able to use this dataset to model specific binding.

### Comparing methods to generate probeset expression values

#### Normalization methods

**Figure 5:**
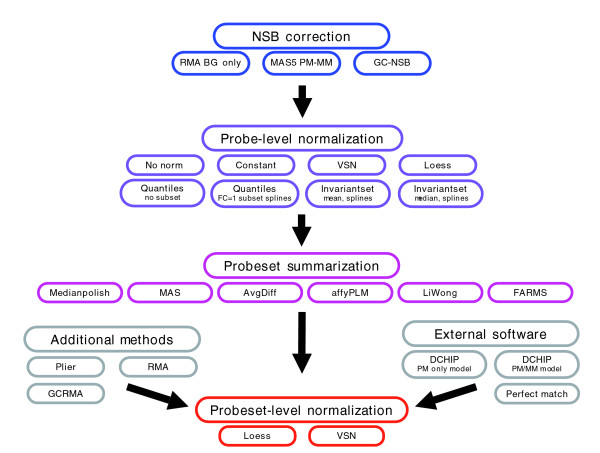
Normalization methods. Diagram of methods used to create probeset expression values. When possible, probe-level normalization used FC = 1 probes as a subset, and all probeset-level normalizations used FC = 1 probesets as a subset. For the normalization methods, additional parameters involve the use of loess or spline to generate a normalization curve. See Materials and methods for more details. BG, background.

Our results emphasize that these sequence-based models are powerful predictors of NSB, and should be applied before further analysis, which agrees with previous observations [22]. Using BioConductor [10], we have combined various background and NSB correction methods to different normalizations and probeset-summarization methods to generate 300 different methods. When possible, we have normalized the probes using the probes within FC = 1 probesets as a subset for normalization; otherwise, all probes were used for normalization. All probeset values were imported into R, and normalized using FC = 1 probesets as a subset. (see Figure 5 and Materials and methods for more details).

Similar to the analysis in [3], we have compared several methods to generate probeset expression values, but we have chosen to evaluate each method based on the following criteria: the estimation of fold changes for FC = 2 probesets, the ability to separate true fold changes from false fold changes, the rate of finding TPs versus the rate of finding FPs, and the difference between calculated *q*-values and true *q*-values. There are too many methods to discuss individually, and we have limited the discussion to groups of methods that have the same NSB correction method and/or same probe summary method, as the choice of NSB correction and probe summary method seem to have the biggest influence on performance.

#### Accuracy and precision

It has been previously observed that background correction "appears to improve accuracy but, in general, worsen precision" [23], and various methods have been put forward to measure accuracy and precision. As the concentration of each transcript is not known but the exact fold change is known in the GoldenSpike experiment, we have chosen the mean log2 fold change for the probesets that can be aligned to transcripts with a two-fold difference (FC = 2) between C and S samples to be a measure of accuracy (mean of 125 probesets with the lowest *P *values as calculated by Cyber-T). As a measure of precision, we have taken 1% of FC = 1 and 1% of empty probesets with the lowest *P *values. Ideally, the log2 fold changes of FC = 2 probesets would be 1 and easily distinguished from fold changes of null probesets, as empty and FC = 1 probesets are expected to have a log2 fold change of zero.

**Figure 6:**
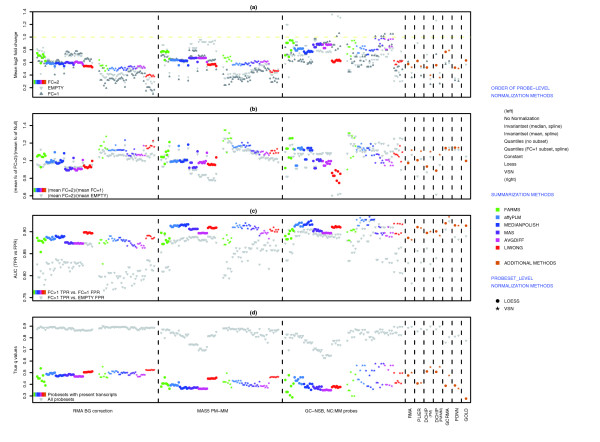
Measures of performance. **(a) **Plot of mean log2 fold changes for FC = 2, empty and FC = 1 probesets for all 300 methods to generate probeset expression values. The mean was generated from the probesets with the lowest Cyber-T *P *values, the lowest 90% for TPs (125 out of 139 for FC = 2) and the lowest 1% for FPs (101 out of 10,104 for empty; 25 out of 2,495 for FC = 1). **(b) **Plot of ratio of mean fold change of TPs (FC = 2) divided by mean fold change of FPs (empty or FC = 1). **(c) **Plot of AUC scores for all probesets and for probesets that can be aligned to present transcripts (FC = 1, FC > 1 and mixed probesets). TPs were FC > 1 probesets and mixed probesets that could be aligned to spiked-in transcripts. All other probesets are true negatives. The plot also includes AUC scores using the FP rate of empty probesets to show which methods work best to reduce FDRs associated with present or absent transcripts. **(d) **Plot of observed FDR (true *q*-value) based on the calculated *q*-values below 0.10 when considering only probesets with present transcripts. To show the contribution of probesets with absent transcripts to FDRs, the plot also includes the observed FDR when all probesets are used.

Methods using GC robust multichip average (RMA) NSB correction (GC-NSB) are the most sensitive and have the highest estimate of FC = 2 but also tend to have the highest estimates of null fold changes. Conversely, methods using RMA background correction are the most specific and have the lowest FC = 2 fold change estimate but also have the lowest null fold change estimates. However, the method of probe summary and probeset-level normalization influences both the estimate of FC = 2 fold changes and the difference between FC = 2 and null fold changes (Figure 6a,b).

#### Performance measured by AUC

While differences between FC = 2 and null probeset fold changes are interesting, it is not a good measure of performance for separating truly differentially expressed genes from FPs. To measure the performance of each method, we calculated the area under the ROC (AUC) using Cyber-T *P *values as predictions. To allow a comparison of AUC measures based on the presence or absence of transcript, we also made two AUC calculations for each method, one using only probesets with 'present' transcripts (FC > 1, FC = 1, and mixed) and one using all probesets (FC > 1, FC = 1, mixed, and empty).

The use of empty probesets results in a drop of AUC performance, especially when only background correction and not NSB correction is used, and this suggests that empty probesets are a more significant source of FPs that bound FC = 1 probesets. The best performing methods for probesets with present transcripts use GC-NSB, affyPLM or medianpolish probe summary and variance stabilization normalization (vsn) probeset-level normalization, but there is little difference between MAS5 PM-MM and GC-NSB methods when the probesets are normalized with the loess method (Figure 6c).

It is also clear in Figure 6c that the GoldenSpike method (called GOLD in the Figures) for combining the statistical analysis of eight different normalization methods [3] does not result in performance gains compared to individual MAS5 PM-MM normalization methods. In fact, the combined statistical analysis tends to under-perform the four individual normalization methods that use medianpolish probe summary.

#### True *q*-values

**Figure 7:**
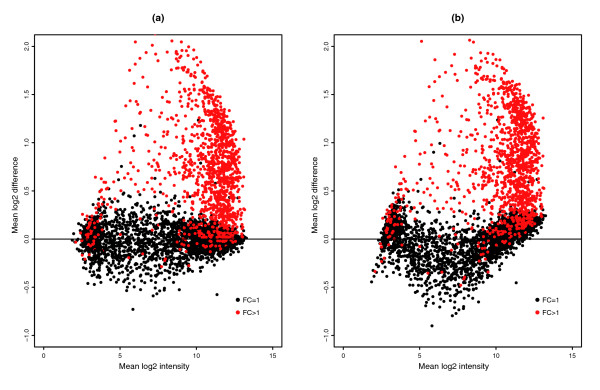
Plot of mean log2 difference versus mean log2 intensity (MA plot) between C and S samples for probesets normalized after loess and vsn. MA plots are for probesets with GC-NSB correction, vsn probe-level normalization, medianpolish probe summary and **(a)** loess or **(b)** vsn probeset-level normalization. The vsn probeset-level normalization method is better than the loess method at separating TPs from FPs (AUC score of 0.916 for loess and 0.928 for vsn), but the null FC = 1 probesets after vsn probeset-level normalization are clearly not centered around zero and the method has more than 25% more FPs with *q*-values below 0.10. Only probesets with present transcripts are shown (FC = 1, black; FC > 1, red).

The AUC performance measure compares only the rate of finding TPs and FPs, and high scoring methods may not be appropriate for a proper analysis of the data. For example, some of the methods with high AUC performance scores give poor estimates of true *q*-values, and users may not be able to distinguish TPs from FPs with a reasonable *P *value or *q*-value because all probesets have very low *P *values (Figure 7). To put the AUC performance measure into context, we calculated *q*-values for every method and compared the actual *q*-values to a calculated *q*-value of 0.10.

In general, methods that correct for NSB tend to have more accurate *q*-values when considering all probesets and only probesets with present transcripts, but the *q*-values generated with all probesets are very poor estimates of the true *q*-value. The method that gave the most accurate measure of *q*-values for probesets with present transcripts was the GoldenSpike method, suggesting that combining statistical analyses might be a method to extract more accurate estimates of *q*-values (Figure 6d).

The AUC performance and true *q*-value comparisons highlight how difficult it is to compare methods to find a 'best method', and it is best left to the user to determine which is the best method in the context of their experiment. However, it is very clear that empty probesets contribute a significant number of FPs and greatly distort *q*-value calculation in the GoldenSpike dataset, and users should gauge the contribution of probesets with absent transcripts to estimated FDRs.

### Performance is dependent on probeset intensity

**Figure 8:**
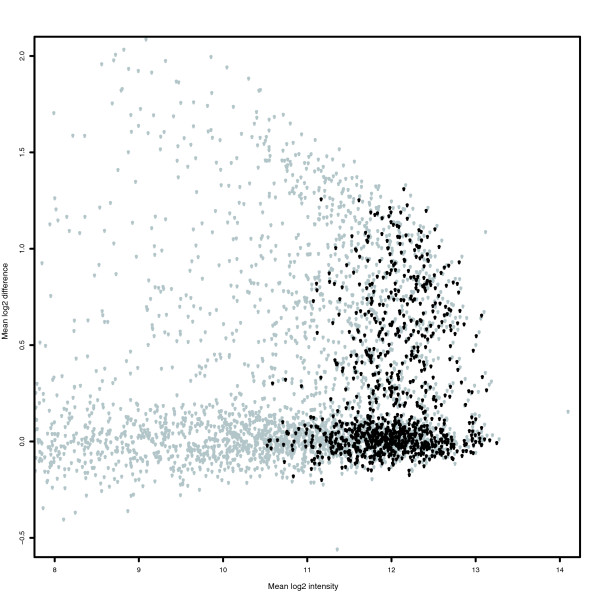
Plot of mean log2 difference versus mean log2 intensity (MA plot) when all probesets are normalized. MA plot for high intensity probesets when all probesets are normalized (grey) and when only the high intensity probesets are used after cel file masking (black). Normalization method is GC-NSB correction, loess probe-level normalization, medianpolish summary and vsn probeset-level normalization.

There has been speculation that the best methods for normalization are dependent on transcript concentrations [23,24]. We have attempted to address the issue by comparing AUC performance (FC > 1 probesets as TPs, all null probesets as FPs) and true *q*-value calculations using probeset intensities as an approximation of transcript concentration. Probesets were classified as unbound, low intensity, medium intensity and high intensity. After removal of unbound probesets, the remaining probesets were placed in categories based on the mean log2 probeset expression value in control replicate samples from a range of methods used to generate probeset expression values and each subset has the same number of FC > 1 probesets. For each category, the cel files were masked to remove all probes that were not part of the probeset category, and probeset expression values were re-calculated. Performance measures were generated for expression values generated from 'masked' cel files and from normalizations using all probes, as there can be subtle but significant differences between the two methods (Figure 8).

#### Unbound probesets

We have defined unbound probesets as probesets that are very unlikely to exhibit specific-binding signal (that is, empty probesets and probesets that are specific for transcripts that are too scarce to be detected). The default settings for the MAS5 present/absent algorithm [7,25] are not stringent enough to identify these probesets, as more than 25% of the probesets classified as having present target transcripts are empty probesets (1,227 out of 4,767 probesets). In addition, there is a significant number of FC = 1 probesets that are not likely to report target transcript specific signal.

**Figure 9:**
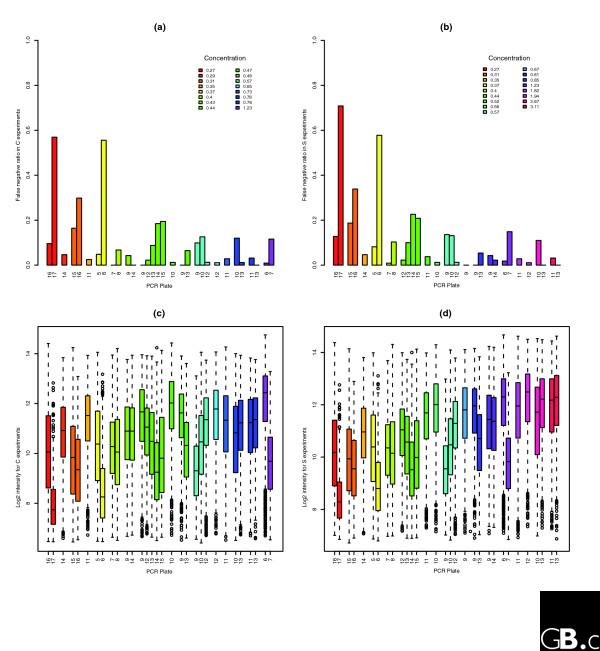
Present/absent calls for each PCR pool. Influence of plate amplification on presence detection and probe brightness. The hybridized cRNA is made of clone pools of given concentrations, and each such pool is made from clones amplified from different plates. The plate numbers are given on the horizontal axis, while the nominal pool concentration is indicated by the color. The top two figures show the false negative ratio (that is, the proportion of probesets falsely called absent) for **(a) **C and **(b) **S samples, as all probesets that can be mapped to a clone should be called present. **(c,d) **The average PM probe intensity of clean probes prior to any normalization or NSB correction. Clean probes match perfectly their target sequence with little promiscuity from other clones' sequences. The figure shows important variations in the amplification efficiency from one plate to the next. The plots show that the proportion of false negatives is highly inversely correlated to the average probe intensity and identifies two 'failed' labeling reactions (plate 17 of concentration 0.27 μg pool, plate 6 of concentration 0.37 μg pool).

A detailed analysis of the present/absent calls indicates two failed labeling reactions, and almost 30% of the FC = 1 and FC > 1 probesets classified as having an absent target transcript come from these two plate-pool mixtures. To make the GoldenSpike dataset, individual PCR-products from 96-well plates were mixed into pools, and the 17 'concentration' pools can be broken down into a further 31 PCR-plate combinations. This means that from a 96-well PCR plate, a subset of PCR products were pooled together, and these subset pools were probably labeled and then combined to make the 17 final pools. The PCR products from plates 16 and 17 make the 0.27 μ*g *pool, but within this pool 60% of the transcripts from plate 17 are absent and less than 10% from plate 16 are absent. Similarly, PCR products from plates 5 and 6 make the 0.37 μ*g *pool, but within this pool 57% of the transcripts from plate 6 are absent and less than 5% from plate 5 are absent. However, within the 1.23 μ*g *pool, less than 1% of PCR products from plate 6 are called absent (Figure 9).

To select probesets that are likely to report only NSB signal, we defined unbound probesets as having a mean log2 probeset expression value below 4 for C and S replicates (after GC-NSB correction). There are 10,322 probesets (9,820 empty, 438 FC = 1, 51 FC > 1 and 13 mixed) classified as unbound probesets, and such a strict cutoff is likely to reflect a realistic level to detect true fold changes. Out of the 300 methods to generate expression values, the highest AUC score is 0.697 and reflects a high cost for finding TPs. A *P *value or *q*-value cutoff designed to find 5 out of the 51 FC > 1 probesets would result in 225 FPs, and 771 FPs to find 10. The unbound probesets are likely to contribute only to FPs, and any statistical significance associated with these probesets should be ignored or the probesets should be removed from any analysis.

#### Low intensity probesets

**Figure 10:**
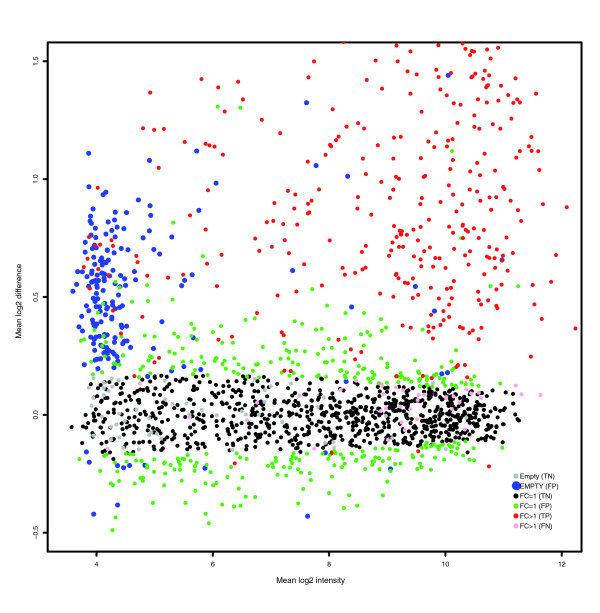
Plot of mean log2 difference versus mean log2 intensity (MA plot) for low intensity probesets. MA plots are for low intensity probesets with GC-NSB correction. In the plots, blue spots represent empty probesets with *q *< 0.1, and these probesets are FPs. Grey symbols represent empty probesets, which are true negatives (TNs). Black symbols represent FC = 1 TNs, and green symbols represent FC = 1 FPs. Red spots represent FC > 1 TPs and pink spots false negatives (FNs).

There are some problems with interpreting AUC and true *q*-value scores of low intensity probesets, as the performance measures are affected by the presence of empty probesets (Figure 10), and AUC and *q*-values have been calculated without empty probesets (see Additional data file 2 for calculations with empty probesets).

**Figure 11:**
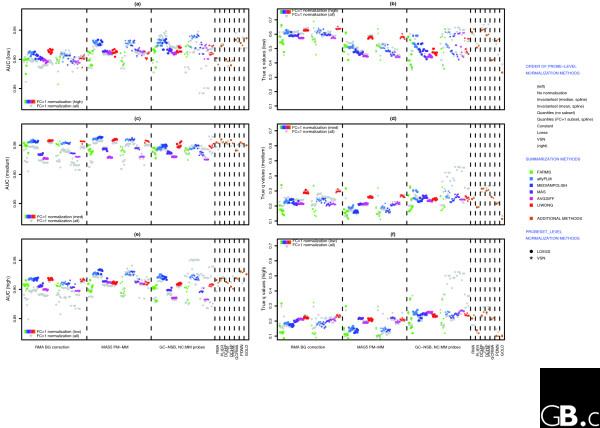
Performance measures for low, medium and high intensity probesets. For each intensity subset of probesets (low, medium and high), 301 methods (Figure 5) were used to generate expression values. All probes that could not be mapped to the subset of interest were masked, and the remaining probesets were normalized with the FC = 1 probesets within each subset. AUC performance scores for **(a) **low, **(c) **medium and **(e) **high intensity probesets were generated for each method using FC > 1 probesets and mixed probesets that can be aligned to spiked-in transcripts as TPs. True negatives were FC = 1 probesets, empty probesets and mixed probesets that can be aligned only to FC = 1 probesets. **(b,d,f) **Plots of observed FDR (true *q*-value) for low (b), medium (d) and high (f) intensity probesets based on the calculated *q*-values below 0.10. AUC and *q*-values were generated for each subset of probesets using a FC = 1 normalization using all probesets with present transcripts (Figure 6). These values show that some methods (for example, GC-NSB with vsn probeset-level normalization) perform very differently depending on the subset of probesets used for normalization.

There is a clear AUC and *q*-value performance gain in using NSB correction (MAS5 PM-MM or GC-NSB), and GC-NSB/(affyPLM or medianpolish probe summary)/vsn probeset-level normalization are the best performing methods, reflecting the performances for all probesets with present transcripts, but for all methods, more than 50% of all probesets with *q*-values below 0.10 are FPs. Typically, there is little difference between performance scores when normalization is done with all probesets or only low intensity probesets, except for some gains in performance for GC-NSB/(affyPLM or medianpolish)/loess probeset-level normalization and loss of performance for some GC-NSB/vsn probeset-level normalization methods when all probesets are used (Figure 11a,b).

#### Medium intensity probesets

The AUC performance of medium intensity probesets is better than low intensity probesets, suggesting that the influence of NSB signal is diminished in medium intensity probesets, and when only medium intensity probesets are normalized, AUC performance for RMA background and MAS5 PM-MM methods improves. The best performance is achieved with MAS5 PM-MM/(affyPLM or medianpolish) probe summary/loess probeset-level normalization methods, and the best performing methods when considering all probesets (GC-NSB/(affyPLM or medianpolish probe summary)/loess probeset-level normalization) perform worse than the equivalent MAS5 PM-MM and RMA background correction methods (Figure 11c).

The *q*-value calculations are also more accurate than low intensity probesets, and MAS5 PM-MM/medianpolish probe summary/(loess or vsn) probeset-level normalization methods have the best combination of AUC and *q*-value performance, but the combined statistical analysis results in the most accurate FDR estimate (Figure 11d).

#### High intensity probesets

The AUC performance of high intensity probesets is less than medium intensity probesets, probably due to the chemical saturation of high intensity probesets. When considering normalization with only high intensity probesets, the PDNN and the MAS5 PM-MM/(affyPLM or medianpolish) probe summary/(loess or vsn) probeset-level normalization methods perform best. When considering normalization of all probesets, GC-NSB/(affyPLM or medianpolish) probe summary/vsn probeset-level normalization methods have the best AUC performance but very poor *q*-value calculations. In general, the *q*-value estimates are more accurate than low and medium intensity probesets, and for the PDNN methods and the combined statistical method, the estimates of 0.10 *q*-values are very accurate and have the best balance between AUC performance and *q*-value accuracy (Figure 11e,f).

#### Performance of high intensity MM probesets

**Figure 12:**
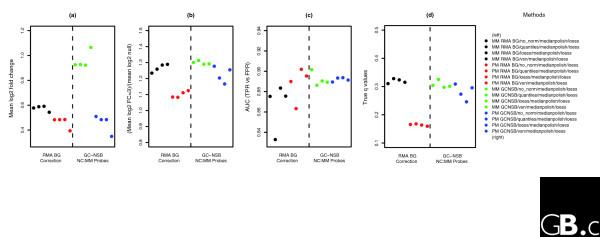
Performance measures for very high intensity MM probesets. **(a) **Plot of mean log2 fold changes for FC = 2 probesets with *q*-values below 0.10 for MM and PM very high intensity probesets. Very high intensity probesets have a mean log2 expression value greater than 12 across all 301 methods used to generate expression values. **(b) **Plot of ratio of mean fold change of TPs (FC = 2) divided by mean fold change of FPs (empty and FC = 1) with *q*-values below 0.10. **(c) **Plot of AUC scores for very high intensity probesets that can be aligned to present transcripts based on Cyber-T *P *values. TPs were FC > 1 probesets and mixed probesets that could be aligned to spiked-in transcripts. All other probesets are true negatives. **(d) **Plot of true *q*-values based on the calculated *q*-values below 0.10. BG, background.

Given that detecting differential expression is restricted at high intensity, we have used MM probes to detect differential expression for the highest intensity probesets (for the top 3% of probesets, the mean log2 expression value for all 300 methods is >12), as confirmation of previous work that showed MM probes can be used to overcome the compression of fold change at very high intensity [24]. For the MM probesets, the estimates of FC > 1 fold changes and the AUC performance after GC-NSB correction and loess probeset-level normalization tends to be better than PM probeset values. However, the accuracy of *q*-value calculations is lower than the equivalent PM probesets (Figure 12).

### The 'alchemy' method

#### Combining methods

It is not practical to subdivide a microarray dataset by probeset intensity and analyze each subset individually with the best performing methods (for example, GC-NSB methods for low intensity probesets, and MAS5 for medium intensity, MAS5 or PDNN for high intensity probesets, and MM probes for very high intensity probesets). Instead, we have chosen 10 methods that have complementary strengths and weaknesses for input into the GoldenSpike statistical method (the 'alchemy' method; see Additional data file 4 for scripts). The benefit of this method is that the combination of four MAS5, four GC-NSB and two PDNN methods to generate expression values outperforms the individual methods and the GoldenSpike method (combined analysis of eight MAS5 PM-MM methods) in AUC performance and true *q*-value tests. The alchemy method also outperforms the GoldenSpike method when only subsets of probesets are used in the statistical analysis (low, medium, and high intensity probesets and probesets that can be aligned to FC = 1 and FC > 1 transcripts). However, the new method does not solve the problem of FPs associated with the differences in NSB of empty and low intensity probesets and results in very poor FDR estimates when considering all probesets.

**Figure 13:**
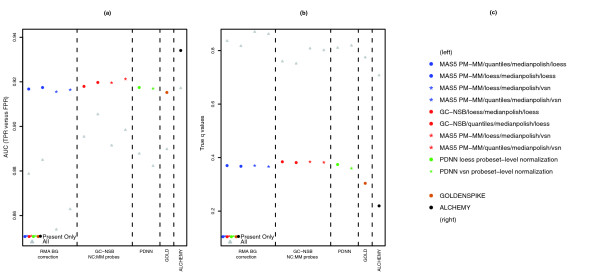
Performance measures for the combined method. **(a)** Plot of AUC scores for true positive rate (TPR) versus false positive rate (FPR) based on Cyber-T *P*-values for individual methods and *q*-values for the combined statistical methods (GOLD and ALCHEMY). True positives were FC > 1 probesets and mixed probesets that could be aligned to spiked-in transcripts. All other probesets are true negatives. Performance measures are based on a subset of probesets with present transcripts. Additional AUC scores were calculated using all probesets to show the influence of probesets with absent transcripts. **(b)** Plot of true *q*-values based on the calculated *q*-values of 0.10. **(c)** Methods used in the alchemy method; the order of processing is NSB correction/probe-level normalization/probe summary/probeset-level normalization.

**Figure 14:**
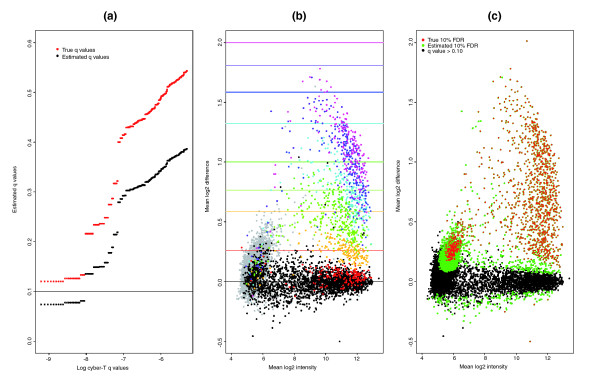
Finding true *q*-values. **(a) **Plot of the percentage of all probesets called 'differentially expressed' that are unbound probesets at various *q*-value cutoffs. **(b) **Mean log2 intensity versus mean log2 difference between C and S replicates for all probesets. Probesets are colored based on their classification. Empty probesets are grey, and those that can be mapped to a transcript are colored by fold change as indicated by the colored lines, which represent the true fold change of each classification. **(c) **Same plot as (b), but probesets are colored by *q*-values. Green symbols represent probesets with calculated *q*-values below 0.10, and red symbols represent probesets with *q*-values < 0.0003 (observed FDR of 10%).

For the GoldenSpike dataset (and probably other datasets), we suggest that unbound probesets are very likely to be FPs, and if there are many unbound probesets with low *q*-values, then the unbound probesets can be removed before normalization or before statistical analysis. For the combined analysis using the 10 methods we suggest, the calculated 0.10 *q*-value is 0.71 when considering all probesets and 0.22 when considering only 'bound' probesets, that is, all probesets are used in normalization but empty probesets were removed from the statistical analysis (Figure 13). Alternatively, the accuracy of *q*-value calculations can be estimated by dividing the number of unbound probesets called positive by the total number of probesets called positive (Figure 14), but the sensitivity of detecting true differential expression is greater when considering only bound probesets. At a true 10% FP rate, there are 114 FPs and 1,019 TPs when using all probesets and 124 FPs and 1,109 TPs when only 'bound' probesets are used for statistical analysis (Table 3).

**Table 3:** True positives and false positives

Category	All probesets	Bound probesets	Total
Empty	102	59	10,104
FC = 1	12	63	2,495
Mixed, FC = 1	1	9	58
Mixed, FC > 1	43	50	69
FC = 1.2	1	9	168
FC = 1.5	123	150	169
FC = 1.7	144	167	179
FC = 2	125	134	139
FC = 2.5	161	166	176
FC = 3	89	90	92
FC = 3.5	166	170	186
FC = 4	167	173	175
TP detected	1,019	1,109	1,353
FP detected	114	124	12,657

The number of TPs and FPs at a true *q*-value of 0.10 for the alchemy method when all probesets are used and when only a subset of probesets with detected transcripts (bound - low, medium and high intensity probesets) are used.

## Conclusion

The knowledge of all transcripts in the GoldenSpike dataset makes it an invaluable resource for understanding the properties of Affymetrix short-oligonucleotide whole genome chips, even though the dataset is far from ideal, with major differences in the amount of labeled cRNA hybridized in C and S samples and the substantial imbalance of transcripts due to the spike-in.

Our analysis of the GoldenSpike dataset extends the performance analysis started by Choe *et al*. [3] to include empirical models and re-enforces the importance of considering probe sequence for NSB estimation. The empirical probe-sequence models have greatly improved our understanding of the Affymetrix GeneChip technology and indicate that we have a good understanding of NSB signal of empty probesets. The probe-sequence based models are significantly better at estimating and correcting NSB signal than the MAS5 PM-MM method, but the improvement in performance of the GC-NSB single-nucleotide model is only evident with empty and low intensity probesets. At medium and high intensity, where NSB signal contributes significantly less to probeset intensity, the MAS5 PM-MM method allows for better detection of spiked-in transcripts, and at high intensity, the PDNN model works well. This suggests that our understanding of the specific binding signal is poor, and any statistical analysis of an individual method is subject to the faults of that method. We suggest a method that combines the GC-NSB, PDNN and MAS5 PM-MM methods. The statistical analysis combining the methods may compensate for the shortcomings of the individual methods and result in better estimates of FDRs. However, a combined statistical analysis is far from ideal, and a better model of DNA-RNA interaction would be preferable.

Hopefully, the GoldenSpike dataset will result in improvements in both the Naef and Zhang models for separating NSB signal from specific binding signal. There are still big improvements needed, especially for modeling the specific binding signal and correcting for chemical saturation, which require additional large-scale datasets in which the concentration of every transcript is known.

## Materials and methods

### Mapping of clones to probes

To achieve a faithful picture of the binding between the clones and the microarray chip, we have mapped all probes' sequences onto the clones' sequences, allowing up to eight alignment errors, either with the PM sequence, or with the MM sequence. In order to perform these alignments, we had to have a careful look at the clone sequences. Out of the 3,870 clones used in the experiments, 3,859 are unique (11 are duplicated on two plates), 3,721 are fully sequenced, and 130 have partial sequence information (120 have 5' and 3' sequences, 7 only 5' sequence and 3 are not attached to any sequence, but are still connected to a known gene) and 8 do not have any kind of sequence information. The fly genome release 4.0 [21] was used to fill the missing sequences for these 130 incomplete clones, against which the partial sequences were aligned to determine the missing parts. This padding was not possible for four clones that have 5' and 3' partial sequences, because the 5' and 3' sequences appeared on very distant genomic locations. Sequence completion was also impossible for one clone with only 5' sequence available, because the only match was on the reverse strand of a known gene. For the other 125 incomplete clones, the sequences were completed with genomic sequences, rather than transcribed sequences, since in most cases there was no known transcript compatible with the clone's partial sequence. In total, there were complete experimental sequences for 3,721 clones, the fly genome was used to complete the sequences of 125 clones, and for 13 clones, no sequence information at all was used, either because they did not have any sequence information in the first place, or because the partial sequence information was in contradiction with the fly genome. To this extent, we were able to get an almost exact picture of the cRNA contents in the samples.

### NSB models

We proceeded by considering all alignments with eight or fewer errors, and we were able to classify PM and MM probes according to the strength of their binding with potentially many different clone sequences. To perform NSB model refinements, we retained empty probes that do not bind any clone with less than seven alignment errors; 54,559 empty probes (25,780 PM and 28,779 MM) matching the criterion above were selected. Finally, 5% of probes at each end (most and least bright) were removed from all computations to reduce the influence of possible outliers, which may arise from contamination by the clones of unknown sequence.

The predicted brightness of our implementation of Naef [17] and Zhang [18] models can be written:

(1) *B *≈ *a *+ *b*exp(***Ax***)

(2) *B *≈ *a *+ *b*/(1 + exp(***w***^*T*^***Ae***))

where *B *is the probe's observed brightness, *a *a constant background and *b *is proportional to the amount of NSB, which is assumed to be identical between all empty probes, but which may depend on the specific experiment, as is the 'optical background' *a*. The other parameters, the relative affinities ***x***, the binding energies ***e ***and the weights ***w ***(necessary because the center of the probe contributes more to the brightness than either end) are assumed to be independent of a particular experiment, and perhaps even valid for different types of GeneChip arrays. The probe sequence is represented by the matrix ***A***, which is such that *A*_*ij *_is 1 when the nucleotide at position *i *is of type *j *(*j *= 1, 2, 3 *or *4 for A, C, G and T) and 0 otherwise.

We treated each chip independently and optimized the models' parameters by a least-squares fit against a subset of 30,000 empty probes, leaving the remaining 24,559 empty probes for cross-validation. The fits were based on log values, to reduce the dynamic range of the observations. This version of the Naef model contains 77 parameters (2 scaling parameters *a *and *b*, and affinities for A, C and G for each of the 25 nucleotide positions; the affinity for T is constrained by the three others). The Zhang model contains only 42 parameters (2 scaling parameters, 16 binding energies and 24 weights, 1 per di-nucleotide position). The ratio of observations to parameters is greater than 350 (Naef model) and 650 (Zhang model), taking into account the trimming of the 5% most and least bright probes for each experiment. The parameter optimization results are summarized in Figure 4.

We also tested the fitted parameters on the cross-validation probes, to ensure that the binding affinities ***x***, or the binding energies ***e ***and their corresponding weights ***w ***were equally able to reproduce intensities from another set of sequences. For this operation, we first put the ***x ***affinities, binding energies ***e ***and weights ***w ***on the same scale for the six experiments. We then used the mean values of those scaled parameters over the six experiments as fixed parameters for the 'scaling fits', allowing for a scaling parameter *s *in the exponent's argument in equations 1 and 2. Therefore, three variables (*a*, *b *and *s*) were optimized against the cross-validation probe intensities. The agreement between the cross-validation intensities and the corresponding modeled values obtained from those fits is very close to the agreements reached by the models where ***x***, ***e***, and ***w ***were refined.

### Normalization

With the exception of PDNN calculations in Perfect Match [26], all of the analysis and graphics produced for this publication used the statistical program R version 2.3.1 [27] and packages within BioConductor [10]. When possible, we broke the process into background/NSB correction (RMA background [8], MAS5 PM-MM [6,7] and GC-RMA [22]), probe-level normalization (loess, constant [6,7], quantiles [8], vsn [28] and invariantset [9]), probe summary into probeset values (medianpolish [8], li-wong [9], tukey-biweight [6,7], farms [29], affyPLM [30], and avgdiff) and probeset-level normalization (vsn and loess). In addition, we calculated probeset values with RMA [8], GC-RMA [22] and probe logarithmic intensity error (PLIER) estimation [31] within BioConductor and with the DNA-Chip Analyzer (dChip) [9] and Perfect Match [26] software. All probeset values were imported into R, and normalized using FC = 1 probesets as a subset.

## Additional data files

The following additional data are available with the online version of this paper. Additional data file 1 is a table of the alignment of clones to probesets. Columns represent (in order): Affy, Affymetrix probeset identifier; Clone, the *Drosophila *Gene Collection clone that aligns to that probeset; C, the concentration pool of that clone in C samples; S, the concentration pool of the clone in S samples; fold, the fold change between S and C samples for the clone; score, alignment score indicating the quality of the alignment (14 = alignment to all 14 probes in probeset); pool, pool number; well, well position in PCR-plate; plate, plate number. Additional data file 2 is a figure showing performance measure for low intensity probesets with and without empty probesets. AUC performance and *q*-value estimates for low intensity probesets when empty probesets are **(a, c) **excluded from the analysis and **(b, d) **included. TPs are FC > 1 probesets and mixed probesets that can be aligned to spiked-in transcripts. True negatives are FC = 1 probesets, empty probesets and mixed probesets that can be aligned only to FC = 1 probesets. Expression values were generated by masking all probes that could not be mapped to the low intensity probesets, re-calculating the probeset expression values and calculating the AUC and *q*-values. AUC and *q*-values were also generated from FC = 1 normalizations using all probesets with present transcripts. See legend for coloring of symbols. Additional data file 3 includes modified GoldenSpike scripts for use in R with BioConductor. Additional data file 4 includes scripts to run the alchemy method in R with BioConductor (requires Additional data file 3)

## Supplementary Material

Additional data file 1 Columns represent (in order): Affy, Affymetrix probeset identifier; Clone, the *Drosophila *Gene Collection clone that aligns to that probeset; C, the concentration pool of that clone in C samples; S, the concentration pool of the clone in S samples; fold, the fold change between S and C samples for the clone; score, alignment score indicating the quality of the alignment (14 = alignment to all 14 probes in probeset); pool, pool number; well, well position in PCR-plate; plate, plate number. Click here for file

Additional data file 2 AUC performance and *q*-value estimates for low intensity probesets when empty probesets are **(a,c) **excluded from the analysis and **(b,d) **included. TPs are FC > 1 probesets and mixed probesets that can be aligned to spiked-in transcripts. True negatives are FC = 1 probesets, empty probesets and mixed probesets that can be aligned only to FC = 1 probesets. Expression values were generated by masking all probes that could not be mapped to the low intensity probesets, re-calculating the probeset expression values and calculating the AUC and *q*-values. AUC and *q*-values were also generated from FC = 1 normalizations using all probesets with present transcripts. See legend for coloring of symbols. Click here for file

Additional data file 3 Modified GoldenSpike scripts for use in R with BioConductor Click here for file

Additional data file 4 Scripts to run the alchemy method in R with BioConductor (requires Additional data file 3) Click here for file

## References

[B1] Binder H, Preibisch S, Kirsten T (2005). Base pair interactions and hybridization isotherms of matched and mismatched oligonucleotide probes on microarrays.. Langmuir.

[B2] Kreil DP, Russell RR (2005). There is no silver bullet-a guide to low-level data transforms and normalisation methods for microarray data.. Brief Bioinform.

[B3] Choe SE, Boutros M, Michelson AM, Church GM, Halfon MS (2005). Preferred analysis methods for Affymetrix GeneChips revealed by a wholly defined control dataset.. Genome Biol.

[B4] Latin Square Data for Expression Algorithm Assessment. http://www.affymetrix.com/support/technical/sample_data/datasets.affx.

[B5] Stapleton M, Carlson J, Brokstein P, Yu C, Champe M, George R, Guarin H, Kronmiller B, Pacleb J, Park S (2002). A *Drosophila *full-length cDNA resource.. Genome Biol.

[B6] Hubbell E, Liu WM, Mei R (2002). Robust estimators for expression analysis.. Bioinformatics.

[B7] Affymetrix Statistical Algorithms Description Document. http://www.affymetrix.com/support/technical/whitepapers/sadd_whitepaper.pdf.

[B8] Irizarry RA, Hobbs B, Collin F, Beazer-Barclay YD, Antonellis KJ, Scherf U, Speed TP (2003). Exploration, normalization, and summaries of high density oligonucleotide array probe level data.. Biostat.

[B9] Li C, Hung Wong W (2001). Model-based analysis of oligonucleotide arrays: model validation, design issues and standard error application.. Genome Biol.

[B10] Gentleman RC, Carey VJ, Bates DM, Bolstad B, Dettling M, Dudoit S, Ellis B, Gautier L, Ge Y, Gentry J (2004). Bioconductor: open software development for computational biology and bioinformatics.. Genome Biol.

[B11] Tusher VG, Tibshirani R, Chu G (2001). Significance analysis of microarrays applied to the ionizing radiation response.. Proc Natl Acad Sci USA.

[B12] Baldi P, Long AD (2001). A Bayesian framework for the analysis of microarray expression data: regularized t-test and statistical inferences of gene changes.. Bioinformatics.

[B13] Storey JD (2002). A direct approach to false discovery rates.. J Roy Stat Soc B Stat Methodol.

[B14] Storey JD (2003). The positive false discovery rate: A Bayesian interpretation and the q-value.. Ann Stat.

[B15] Storey JD, Tibshirani R (2003). Statistical significance for genomewide studies.. Proc Natl Acad Sci.

[B16] Dabney A, Storey J (2006). A reanalysis of a published Affymetrix GeneChip control dataset.. Genome Biol.

[B17] Naef F, Magnasco MO (2003). Solving the riddle of the bright mismatches: Labeling and effective binding in oligonucleotide arrays.. Phys Rev E Stat Nonlin Soft Matter Phys.

[B18] Zhang L, Miles MF, Aldape KD (2003). A model of molecular interactions on short oligonucleotide microarrays.. Nat Biotechnol.

[B19] Lehmann E (1997). Testing Statistical Hypotheses.

[B20] McGee MC, Chen Z (2006). New spiked-in probe sets for the affymetrix hgu-133a Latin Square experiment.. COBRA Preprint Series.

[B21] Drysdale RA, Crosby MA, Consortium F (2005). FlyBase: genes and gene models.. Nucleic Acids Res.

[B22] Wu Z, Irizarry RA (2005). Stochastic models inspired by hybridization theory for short oligonucleotide arrays.. J Comput Biol.

[B23] Irizarry RA, Wu Z, Jaffee HA (2006). Comparison of Affymetrix GeneChip expression measures.. Bioinformatics.

[B24] Naef F, Socci ND, Magnasco M (2003). A study of accuracy and precision in oligonucleotide arrays: extracting more signal at large concentrations.. Bioinformatics.

[B25] Liu Wm, Mei R, Di X, Ryder TB, Hubbell E, Dee S, Webster TA, Harrington CA, Ho Mh, Baid J, Smeekens SP (2002). Analysis of high density expression microarrays with signed-rank call algorithms.. Bioinformatics.

[B26] Perfect Match. http://odin.mdacc.tmc.edu/~zhangli/PerfectMatch/.

[B27] R Development Core Team (2005). R: A Language and Environment for Statistical Computing.

[B28] Huber W, von Heydebreck A, Sultmann H, Poustka A, Vingron M (2002). Variance stabilization applied to microarray data calibration and to the quantification of differential expression.. Bioinformatics.

[B29] Hochreiter S, Clevert DA, Obermayer K (2006). A new summarization method for affymetrix probe level data.. Bioinformatics.

[B30] Bolstad B (2004). Low level analysis of high-density oligonucleotide array data: background, normalization and summarization.. PhD thesis.

[B31] PLIER Technical Note. http://www.affymetrix.com/support/technical/technotes/plier_technote.pdf.

