# Supplementary material for: Estimation and correction of non-specific binding in a large-scale spike-in experiment
Source: Genome Biol. 2007 Jun 26;8(6):R126. doi: 10.1186/gb-2007-8-6-r126 (PMC2394775; doi:10.1186/gb-2007-8-6-r126)
Supplement: Additional data file 2 — AUC performance and q-value estimates for low intensity probesets when empty probesets are (a,c) excluded from the analysis and (b,d) included. TPs are FC > 1 probesets and mixed probesets that can be aligned to spiked-in transcripts. True negatives are FC = 1 probesets, empty probesets and mixed probesets that can be aligned only to FC = 1 probesets. Expression values were generated by masking all probes that could not be mapped to the low intensity probesets, re-calculating the probeset expression values and calculating the AUC and q-values. AUC and q-values were also generated from FC = 1 normalizations using all probesets with present transcripts. See legend for coloring of symbols. [file gb-2007-8-6-r126-S2.pdf]

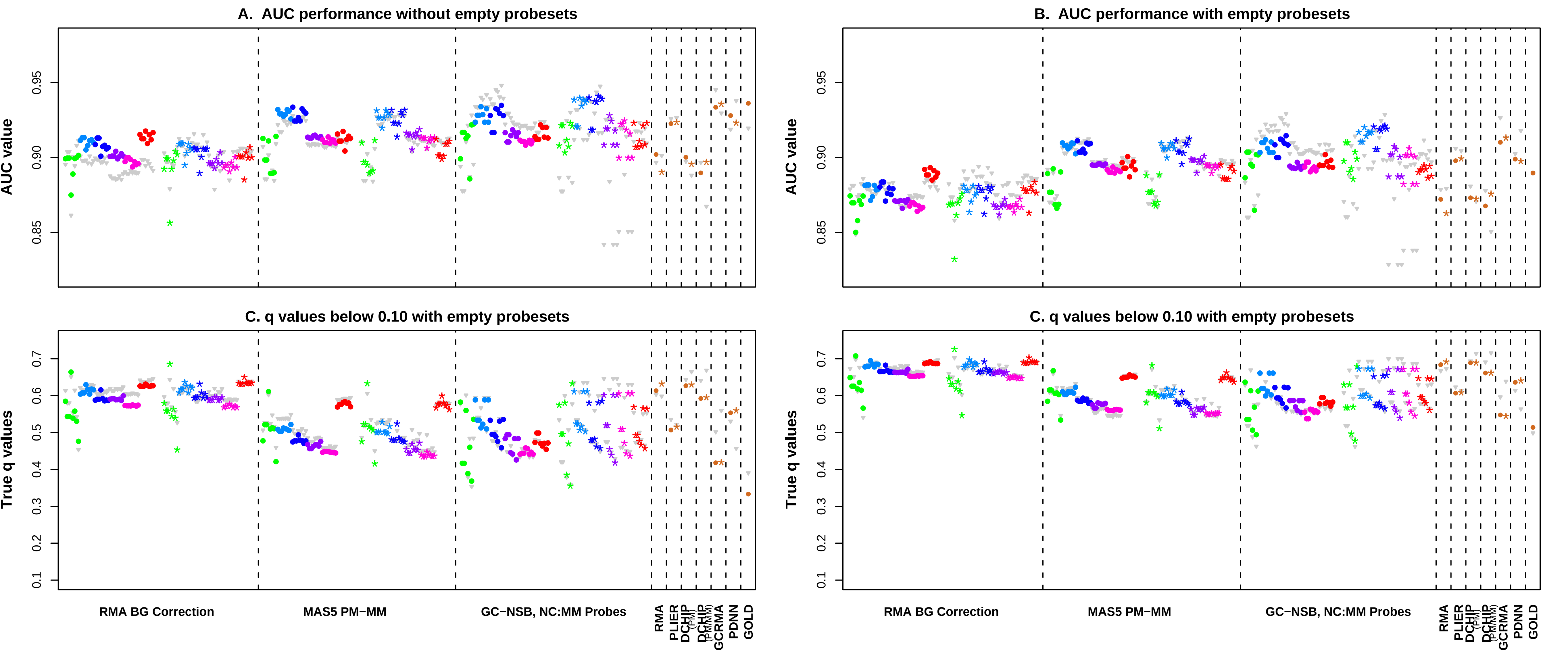
A. AUC performance without empty probesets
B. AUC performance with empty probesets
C. q values below 0.10 with empty probesets
C. q values below 0.10 with empty probesets
AUC value
True q values
0.85
0.90
0.95
0.1
0.2
0.3
0.4
0.5
0.6
0.7
RMA BG Correction
MAS5 PM−MM
GC−NSB, NC:MM Probes
RMA
PLIER
DCHIP (PM)
DCHIP (PM/MM)
GCRMA
PDNN
GOLD
